# Analysis of neural networks for routine classification of sixteen ultrasound upper abdominal cross sections

**DOI:** 10.1007/s00261-023-04147-x

**Published:** 2024-01-12

**Authors:** Alistair Lawley, Rory Hampson, Kevin Worrall, Gordon Dobie

**Affiliations:** 1 Faculty Electronic and Electrical Engineering, University of Strathclyde, Glasgow, UK; 2 Faculty of Engineering, University of Glasgow, Glasgow, UK

**Keywords:** Ultrasound, Classification, Abdominal screening, Machine learning

## Abstract

**Purpose:**

Abdominal ultrasound screening requires the capture of multiple standardized plane views as per clinical guidelines. Currently, the extent of adherence to such guidelines is dependent entirely on the skills of the sonographer. The use of neural network classification has the potential to better standardize captured plane views and streamline plane capture reducing the time burden on operators by combatting operator variability.

**Methods:**

A dataset consisting of 16 routine upper abdominal ultrasound scans from 64 patients was used to test the classification accuracy of 9 neural networks. These networks were tested on both a small, idealised subset of 800 samples as well as full video sweeps of the region of interest using stratified sampling and transfer learning.

**Results:**

The highest validation accuracy attained by both GoogLeNet and InceptionV3 is 83.9% using transfer learning and the large sample set of 26,294 images. A top-2 accuracy of 95.1% was achieved using InceptionV3. Alexnet attained the highest accuracy of 79.5% (top-2 of 91.5%) for the smaller sample set of 800 images. The neural networks evaluated during this study were also successfully able to identify problematic individual cross sections such as between kidneys, with right and left kidney being accurately identified 78.6% and 89.7%, respectively.

**Conclusion:**

Dataset size proved a more important factor in determining accuracy than network selection with more complex neural networks providing higher accuracy as dataset size increases and simpler linear neural networks providing better results where the dataset is small.

## Introduction

There has been a significant increase in demand for diagnostic medical imaging [1], with some healthcare providers seeing an average annual increase of demand of ~ 5% for ultrasound [2]. Ultrasound has seen widespread adoption throughout healthcare due to the broad range of applications and accessibility of ultrasound equipment, especially in mid to low-income countries where access to other modalities can be limited [3, 4]. Meeting this increased demand for ultrasound scans is a complex problem, not only is there a chronic shortage of skilled sonographers [5, 6] but the collection of ultrasound is a highly manual process of the sonographer directly pressing the probe against the patient and as such relies heavily on the attentiveness, knowledge, and experience of the individual sonographer [7] to ensure a good result. This manual aspect leads to an increased risk of workplace injuries such as repetitive strain injuries within the sonographic workforce [8, 9]. The use of deep learning offers a potential solution by reducing the time taken for each ultrasound procedure by automating the capture of relevant cross-sectional imagery, ensuring adherence to protocol, improving workflow and patient comfort. For this to become reality a large ultrasound protocol, that is representative of clinical workflow much be benchmarked to gauge the response of current deep learning technologies.

Image classification is a fundamental component of medical machine learning image research, of which deep learning is an increasingly popular subject of interest [10, 11]. Despite being one of the most widely used medical imaging modalities in the world, ultrasound has seen comparatively little interest from deep learning research in comparison to radiography (Xray), computer tomography (CT) and nuclear magnetic resonance imaging (MRI) [12]. This is partly due to the fact that there are very few clinical ultrasound datasets available in comparison to other modalities. Ultrasound is produced by measuring the reflected ultrasound waves detected by a small piezoelectric array within the ultrasound probe [13], such images are typically two-dimensional, low contrast, and subject to interference such as attenuation and shadowing that can hinder classification even for experienced sonographers [14, 15].

Machine learning has already been successfully applied to many classification tasks within medical diagnostic ultrasound such as cancer diagnosis [16, 17], thyroid nodules [18, 19], liver anomalies [20, 21], spine [22] and cardiac cross sections [23–25]. Previous studies examining classification of abdominal cross sections with machine learning are limited. Cheng & Malhi [26] proved the effectiveness of transfer learning using the ImageNet challenge dataset [27] with the successful classification of 11 standard ultrasound cross sections attaining accuracies of 77.3% using CaffeNet and 77.9% for VGGNet both of which exceeded the 71.7% accuracy achieved by a radiologist. Xu et al. [28] examined classification of 11 ultrasound abdominal cross sections as part of a wider study on landmark detection, the Single-task learning (STL) ResNet-50 attained an accuracy of 81.22% in comparison to the radiologist who achieved 78.87%. Reddy et al. [29], tested a number of neural networks on 6 visually distinct abdominal cross sections achieving an accuracy of 98.77% using a ResNet-50.

This study examines 16 upper abdominal cross sections as defined by the Japanese abdominal screening protocol [30]. This protocol was chosen due to its overlapping coverage of the upper abdomen, which would underline and potential difficulties applying deep learning to complex ultrasound abdominal protocols. While the Japanese abdominal screening protocol includes pelvic and bladder scans, these were excluded from this study to focus on the upper abdomen.

## Materials and methods

### Ultrasound data acquisition

**Fig. 1:**
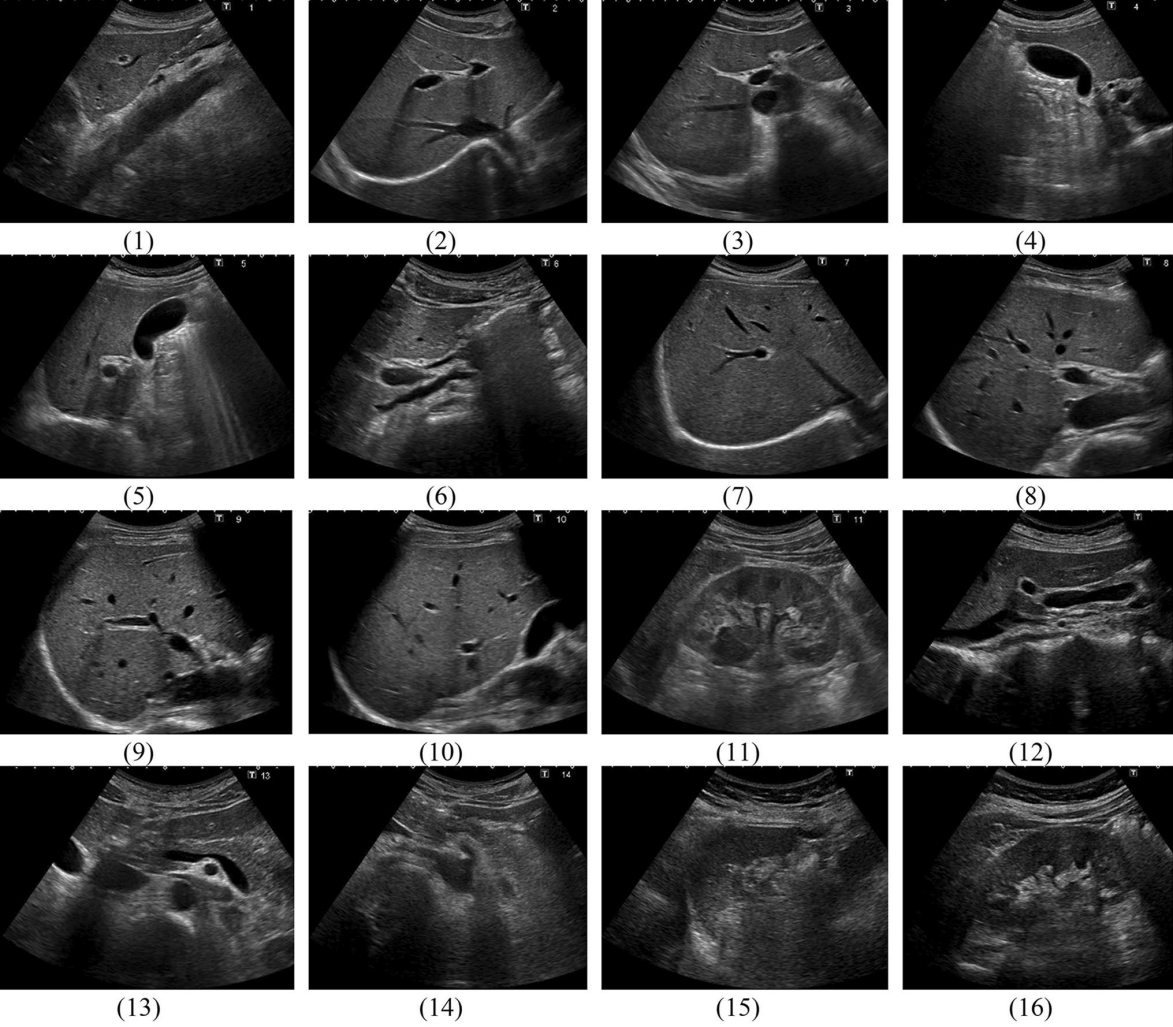
Example of the 16 upper abdominal ultrasound cross sections: 1. Epigastric sagittal (liver/aorta), 2. Epigastric horizontal (hepatic vein), 3. Right Epigastric oblique (horizontal portal vein), 4. Right Subcostal (gallbladder), 5. Right hypochondrium vertical (gallbladder), 6. Right hypochondrium vertical (bile duct), 7. Right subcostal (liver), 8. Right intercostal (liver), 9. Right intercostal (liver), 10. Right intercostal (liver), 11. Right intercostal (kidney), 12. Epigastric vertical (bile duct/pancreas), 13. Epigastric horizontal (pancreas), 14. Epigastric oblique (Pancreas), 15. Spleen, 16. Left intercostal (kidney)

The ultrasound data were captured using a Canon TUS-AI800 [31] using a curved linear array, with each of the 16 cross sections (examples of which are displayed in Fig. 1.) classified at the time of capture by a single experienced sonographer. While the data are anonymous, acceptance criteria was that participants be of adult age with no underlying pathology detected by the sonographer that may influence the study results at time of recording. The sonographer strictly adhered to the standardised capture method defined by the Japanese society of sonographers [30], starting the scan in the location defined within the method and progressively sweeping through the region of interest ensuring complete coverage of the defined target anatomy. The ultrasound data were recorded as a stream of 8-bit greyscale images of varying length (between 14 and 46 s), these sequences were effectively raw ultrasound images and contained no text or graphical annotation from the User Interface. These were then stored in a DICOM format [32] and anonymised before being provided for use in this work.

The dataset consists of 64 patient studies with 16 recorded anatomical cross sections each for a total of 1024 image streams and a total of 33,093 individual images. These patient studies were split 50/14 (approximately 80/20 split) between training and test sets, both training and test sets were resampled at the patient level for each training run for cross validation purposes, although this significantly reduces the pool of possible test images it was done to ensure no data leakage that could artificially inflate results.

Two training sets were produced alongside a single test set as reported in Table 1. The first training set was produced to provide a balanced, idealised dataset by defining a single image frame (an example of which can be seen in Fig. 1.) from each set of cross sectional sweeps for a total of 800 images, this was done to simplify the problem space, while in many cases a sonographer must move the probe to fully visualise the region of interest, reduction to a single ideal cross section provides the neural network with the most opportunity to make the correct prediction. The second training set takes into account the entire sonographic sweep and as such essentially consists of multiple short videos centred on the correct region of interest during examination and is made up of 26,294 images, this data contain significant repetition, minor deviations such as changes in attenuation, shadowing, natural physiological changes, and the slight movements of the patient and sonographer that occur naturally during clinical examination. This provides a more realistic training set but also significantly increases the complexity of classification. The test set consists of 224 images with each of the 16 cross sections represented by 14 precise images. Those images and videos corresponding to the test set were excluded from all training datasets.

**Table 1:** Identified upper abdominal cross section categories in training and test sets

Upper abdominal cross section	Training Set 1	Training Set 2	Test Set
1. Epigastric sagittal scan: Liver/aorta	50 (6.3%)	1478 (5.6%)	14 (6.3%)
2. Epigastric horizontal scan to right subcostal scan: Hepatic vein	50 (6.3%)	1722 (6.5%)	14 (6.3%)
3. Right Epigastric oblique scan: Horizontal portal vein	50 (6.3%)	1605 (6.1%)	14 (6.3%)
4. Right Subcostal scan: Gallbladder	50 (6.3%)	1545 (5.9%)	14 (6.3%)
5. Right hypochondrium vertical scan: Gallbladder	50 (6.3%)	1539 (5.9%)	14 (6.3%)
6. Right hypochondrium vertical to oblique scan: Bile duct	50 (6.3%)	1575 (6%)	14 (6.3%)
7. Right subcostal scan: Liver	50 (6.3%)	1528 (5.8%)	14 (6.3%)
8. Right intercostal upper scan: Liver	50 (6.3%)	1558 (5.9%)	14 (6.3%)
9. Right intercostal mid scan: Liver	50 (6.3%)	1670 (6.4%)	14 (6.3%)
10. Right intercostal lower scan: Liver	50 (6.3%)	1609 (6.1%)	14 (6.3%)
11. Right intercostal scan: Right kidney	50 (6.3%)	1516 (5.8%)	14 (6.3%)
12. Epigastric vertical scan: Extrahepatic bile duct/pancreas	50 (6.3%)	1717 (6.5%)	14 (6.3%)
13. Epigastric horizontal scan: Pancreas	50 (6.3%)	1886 (7.2%)	14 (6.3%)
14. Epigastric oblique scan: Pancreas	50 (6.3%)	1972 (7.5%)	14 (6.3%)
15. Left intercostal scan: Spleen	50 (6.3%)	1759 (6.7%)	14 (6.3%)
16. Left intercostal scan: Left kidney	50 (6.3%)	1615 (6.1%)	14 (6.3%)
Total	800	26,294	224

As the transfer learning neural networks are trained on 3 channel RGB images, the single channel greyscale images were duplicated into three channels during the process to convert the image into tensors of size 299 × 299. Results from version 1 and version 3, as well as the other highlighted architectures, are analysed in this work. The full image was used with no cropping or adjustment beyond minor contrast normalisation using the standard method provided in Pytorch in order to ensure standardisation across the imagery. No additional de-speckling, image filtering or post processing was performed post capture, this was to ensure that imagery was representative of the classification task required of sonographers.

### Neural network architectures

The experiment was performed on a computer with an Intel CPU with a clock speed of 2.4 GHz and a Nvidia 20 series GPU using the Pytorch framework [33] and Cuda toolkit (version 11.6). As with previous literature [26, 28, 29] publicly available neural networks pre-trained on the ImageNet challenge dataset [27] were used as the basis for transfer learning. The neural networks architectures chosen for this experiment can be classified by the principles behind their design. These being two linear convolutional neural networks (Alexnet [34, 35], VGGNet [36]), five residual networks (ResNet-18, 32, 50, 101, 152) [37], and two inception networks (GoogLeNet (Inception V1) [38] and InceptionV3 [39]). A summary of the exact number of layers and parameters used by the neural networks in this study is provided in Table 2. These neural networks were chosen as typical examples of their respective architectures, with five residual networks evaluated to test how the depth of residual network effects network response to ultrasound data. Three training procedures were used: transfer learning using dataset 1, transfer learning using dataset 2, and a baseline using only training dataset 2 without pre-trained transfer learning weightings being applied at initialisation. Training used the ADAM optimiser [40] with an initial learning rate of 1 × 10^–4^ with the learning rate degrading every 5 steps, over 20 epochs. Each network was trained 20 times with the training and test sets resampled for each training run in order to benchmark performance while reducing performance variation from any single training run. The final layer of each neural network was adjusted from 1000 to 16 in order for the neural networks to perform the required classification task, no additional changes were made from the standard network architecture used for ILSVR Challenge [27].

**Table 2:** Summary of neural network shape and parameters

Model	Method	Convolution	Fully connected	Parameters
Alexnet	Linear	5	3	57,069,392
VGG16	Linear	13	3	134,326,096
GoogleNet	Inception	22	1	11,996,288
InceptionV3	Inception	48	1	25,145,048
ResNet-18	Residual	18	1	11,184,720
ResNet-34	Residual	34	1	21,292,880
ResNet-50	Residual	50	1	23,540,816
ResNet-101	Residual	101	1	42,532,944
ResNet-152	Residual	152	1	58,176,592

## Results

The results for highest single neural network accuracy of the nine neural networks (as shown in Table 3) show that the Inception architecture achieved the highest accuracies on the test set for both transfer learning with dataset 2 and the Baseline, with GoogLeNet (InceptionV1) and InceptionV3 attaining the top result of 83.93% for dataset 2, with inceptionV3 attaining 79.91% and GoogLeNet 77.68% for the Baseline. Linear neural network architectures attained the highest results for dataset 1 with Alexnet achieving 79.46% and 77.23% for VGG16.

**Table 3:** Highest accuracy achieved after 20 epochs from nine neural networks over 20 training runs

	Alexnet (%)	VGG16 (%)	GoogLeNet (%)	InceptionV3 (%)	ResNet 18 (%)	ResNet 34 (%)	ResNet 50 (%)	ResNet 101 (%)	ResNet 152 (%)
Baseline Accuracy	69.20	70.09	77.68	79.91	75.06	73.66	73.21	71.88	71.43
Dataset 1 Accuracy	79.46	77.23	62.05	71.88	67.41	73.21	73.21	70.98	70.54
Dataset 2 Accuracy	80.80	82.59	83.93	83.93	83.04	83.48	83.48	82.14	83.04

**Fig. 2:**
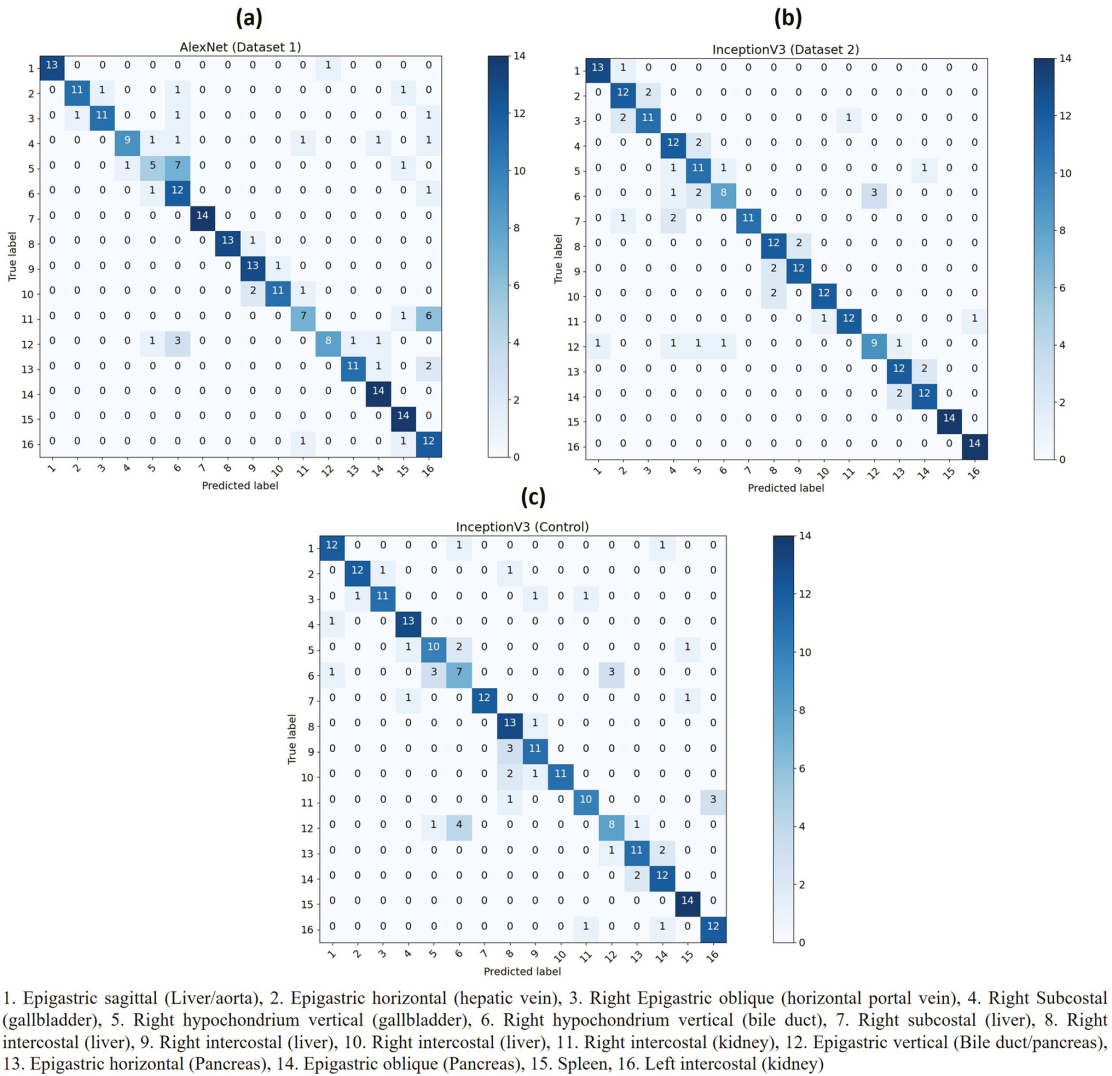
Confusion Matrix for top performing neural networks: **a** Alexnet Dataset 1, **b** InceptionV3 Dataset 2, **c** InceptionV3 Baseline Control Dataset

The confusion matrix in Fig. 2 confirms that the largest misclassification errors are: between cross sections within close proximity such as cross Sects. 8, 9 and 10 which focus on the liver; where anatomical structures overlap such as in cross Sects. 5 and 6 which focus on vertically oriented biliary system, as well as 6 and 12 which the bile duct is a significant landmark; and differentiating between the kidneys in cross Sects. 11 and 16.

Top-2 accuracy results (shown in Table 4) continue the trend with InceptionV3 attaining the highest top-2 accuracy of 92.86% for Baseline with the second-best result being GoogLeNet with 90.18%. The linear architectures attained the highest top-2 accuracy in dataset 1 with Alexnet attaining 91.52% and 90.18% for VGG16. InceptionV3 also achieved the highest top-2 for dataset 2 at 95.09% but ResNet 18, 34 and 50 jointly attained the second-best result of 94.64%. The neural networks with the highest overall accuracy did not correspond to that of the highest top-2 accuracy. Those that did match were ResNet101, ResNet152 for Baseline; Alexnet, VGG16 and GoogLeNet corresponded for Dataset 1 and VGG16, ResNet34 and ResNet50 for Dataset 2.

**Table 4:** Highest top-2 accuracy attained accuracy after 20 training runs

	Alexnet (%)	VGG16 (%)	GoogLeNet (%)	InceptionV3 (%)	ResNet 18 (%)	ResNet 34 (%)	ResNet 50 (%)	ResNet 101 (%)	ResNet 152 (%)
Baseline Top-2	86.16	83.04	90.18	92.86	87.95	87.50	89.29	87.05^a^	87.05^a^
Dataset 1 Top-2	91.52^a^	90.18^a^	79.46^a^	88.84	84.38	88.84	87.05	86.16	87.05
Dataset 2 Top-2	92.86	93.75^a^	94.20	95.09	94.64	94.64^a^	94.64^a^	94.20	93.75

^a^Accuracy and Top-2 attained from same neural network model

The testing algorithm included category specific accuracy results (shown in Table 5) allowing for a deeper examination of the strengths and weaknesses of ultrasound plane categorisation. When examining the plane specific categorisation results from the InceptionV3 neural network trained from Dataset 2 it was possible to correctly categorise the right kidney plane 78.57% and the left kidney plane 89.71% of the time suggesting sufficient visual information is available to achieve successful classification. When examining the overall performance of transfer learning with Dataset 2 (from Table 5), the cross sections with the lowest accuracy were plane 6 (Right hypochondrium vertical to oblique scan: Extrahepatic bile duct) with an average accuracy of 64.29%, and Plane 12 (Epigastric vertical scan: Extrahepatic bile duct/pancreas) with an average of 67.46%. These cross sections see the highest error in each of the three exampled confusion matrixes, this is likely due to intersecting anatomical structures within the plane classifiers.

**Table 5:** Accuracy of individual cross sections: highest single neural network accuracy trained using dataset 2

Cross section	Alexnet (%)	VGG16 (%)	GoogLeNet (%)	InceptionV3 (%)	ResNet 18 (%)	ResNet 34 (%)	ResNet 50 (%)	ResNet 101 (%)	ResNet 152 (%)
1. Epigastric sagittal: Liver/aorta	92.86	100	100	100	100	100	100	100	100
2. Epigastric horizontal: Hepatic vein	78.57	92.86	85.71	78.57	92.86	85.71	92.86	85.71	92.86
3. Right Epigastric oblique: Horizontal portal vein	92.86	78.57	78.57	78.57	78.57	78.57	85.71	85.71	85.71
4. Right Subcostal: Gallbladder	71.43	64.29	78.57	78.57	71.43	85.71	71.43	64.29	78.57
5. Right hypochondrium vertical: Gallbladder	71.43	71.43	71.43	78.57	71.43	85.71	71.43	78.57	71.43
6. Right hypochondrium vertical: Bile duct	71.43	50.00	64.29	71.43	64.29	57.14	64.29	57.14	78.57
7. Right subcostal: Liver	85.71	85.71	78.57	85.71	92.86	78.57	85.71	78.57	71.43
8. Right intercostal: Liver	78.57	85.71	100	85.71	92.86	92.86	85.71	78.57	100
9. Right intercostal: Liver	92.86	85.71	85.71	85.71	78.57	92.86	85.71	78.57	92.86
10. Right intercostal: Liver	78.57	85.71	85.71	92.86	78.57	85.71	85.71	85.71	71.43
11. Right intercostal: Right kidney	64.29	78.57	78.57	78.57	85.71	78.57	92.86	85.71	64.29
12. Epigastric vertical: Bile duct/pancreas	57.14	71.43	71.43	78.57	64.29	64.29	64.29	71.43	64.29
13. Epigastric horizontal: Pancreas	85.71	100	85.71	78.57	92.86	100	100	92.86	100
14. Epigastric oblique: Pancreas	85.71	78.57	85.71	85.71	71.43	57.14	71.43	71.43	71.43
15. Left intercostal: Spleen	100	100	100	100	100	100	100	100	100
16. Left intercostal: Left kidney	85.71	92.86	92.86	85.71	92.86	92.86	78.57	100	85.71
Total	80.80	82.59	83.93	83.93	83.04	83.48	83.48	82.14	83.04

Examining the variation in training outcome between the 20 runs (detailed in Table 6), shows that in most cases using the full dataset and transfer learning (dataset 2) reduced variation in training result with the exception of ResNet-18 with a variation of 13%. Inception based neural networks achieved the lowest variance with GoogLeNet had the smallest variation of 6% and InceptionV7 achieving 7%. Alexnet achieved the highest accuracy for dataset 1 but there was notable variance in the result of 22%, GoogLeNet achieved the poorest overall accuracy but also smallest variance.

**Table 6:** Variance in training outcome based on the standard deviation for neural networks over 20 runs

Model	Dataset 1 (%)	Dataset 2 (%)	Baseline (%)
Alexnet	22	7	11
VGG-16	21	8	10
ResNet-18	19	13	8
ResNet-34	21	9	15
ResNet-50	25	10	13
ResNet-101	26	10	15
ResNet-152	22	10	13
GoogLeNet	9	6	14
InceptionV3	13	7	10

## Discussion

This study examined the effectiveness of transfer learning for a small ultrasound abdominal cross-sectional dataset, providing comparative accuracy data for a larger number of neural network architectures on standard abdominal cross sections than has been previously studied. This will serve both to aid selection of neural networks in future, but also further highlights the potential uses and difficulties of utilising deep learning for identifying and classifying upper abdominal cross sections. While the size of the test set is small, this study provides a benchmark as to expected performance of neural networks for medical ultrasound classification tasks on 16 upper abdominal cross sections. It has been possible to compare traditional learning using a relatively small medical ultrasound dataset of just 26,294 uneven non-ideal samples, with two transfer learning experiments using the ILSVRC data set [27], one leveraging a balanced idealised sample set of just 800 and the other using transfer learning the augment the entire dataset. Optimisation of techniques for convolutional neural networks has seen many improvements with traditional machine learning using the InceptionV3 neural network able to achieve a result of 79.91%, just 4.02% lower than the highest result achieved by transfer learning in only 20 epochs. Furthermore, with transfer learning it was possible to use just 800 samples to train a network to attain an accuracy of 79.46%, just 4.47% from the best result from the larger dataset 2. The use of transfer learning and the complete dataset produced the best result of 83.93% with the result being shared by both Inception neural networks tested.

The residual network architecture did not produce the highest accuracy models (as seen in Table 3) but does improve in accuracy as the size dataset increases with results for dataset 2 showing accuracies typically within 1% of the highest result. As previously discussed, residual mapping should have allowed each of the ResNet models to attain similar accuracy results with some variation expected from training randomisation. ResNet 34 and 50 both achieved the highest accuracies of 73.21% for dataset 1 and 83.48% for dataset 2 but ResNet18 achieved the highest baseline accuracy of 75.06%. The difference between highest and lowest performing ResNet neural network was 3.63% for the Baseline, 5.80% for dataset 1, and 1.34% for dataset 2, suggesting that residual mapping struggled with the smaller datasets which would also partially account for the subsequent drop off in accuracy in the larger ResNet-101 and 152 models.

Despite the use of 16 upper abdominal cross sections with many overlapping anatomical structures the top performing neural networks (Table 5) achieved an average overall accuracy of 82.94% with greatest error occurring between cross sections containing overlapping identifiers. Where the top-2 accuracy is considered, the neural networks studied achieved an accuracy between 79.46% and 95.09% with the top 10 models being within 2.2% accuracy. The high top-2 accuracy and confusion matrix (Fig. 2) suggests that while a positive prediction was being made the similarities between cross sections played a major role in reducing accuracy as the majority of errors correspond with cross sections containing the same anatomical structures such as right liver cross Sects. 8, 9 and 10, cross Sects. 6 and 12 which both contain the extrahepatic bile duct as the main region of interest and differentiating the left and right kidneys in cross Sects. 11 and 16.

The variation in accuracy recorded suggests that larger neural networks benefitted from the larger dataset (dataset 2) and transfer learning the most, ResNet-101 and ResNet-152 displayed notably lower per-plane accuracy results for dataset 1, improved accuracy results for the baseline and then most improved with the addition of transfer learning (dataset 2). While variance itself is less relevant than accuracy as a training metric, neural networks with a smaller variance are more likely to achieve a result closer to the highest accuracy in fewer iterations. Transfer learning can significantly improve accuracy but is no substitute for data. While dataset 1 was too small to provide sufficient information for traditional machine learning to provide a useful result it was capable of producing surprisingly accurate results rivalling the larger baseline dataset and warrants further examination of the effect of ultrasound sample size on neural network learning and generalisation in future works. This study also suggests that the number of layers was less important than dataset size when performing upper abdominal ultrasound plane classification with the difference in accuracy of neural networks for dataset 2 being just 2.2%. Transfer learning also significantly improved neural network accuracy with the larger dataset, when comparing dataset 2 with the baseline, the per-plane training variance is noticeably reduced with the addition of transfer learning along with a significant improvement in accuracy. While dataset size was a more significant factor in reducing variance and increasing accuracy, transfer learning allows for significant improvements to ultrasound plane classification accuracy where the data is sufficient for the number of parameters in the neural network used.

While there are limitations to the amount of direct comparison that can be made as previous studies used different cross sections, it is possible to highlight a number of trends when classifying abdominal ultrasound data. As seen in Table 7, comparing the accuracy results of transfer learning on dataset 2, the overall the results of this study are in line with those of previous studies. Smaller networks such as Alexnet achieved an accuracy result just 3.13% lower than the highest accuracy network, show significant potential to classify ultrasound cross sections, CaffeNet (a variant of Alexnet) achieved just 0.6% lower than the significantly larger VGGNet used in Cheng and Malhi [26], and 3.5% lower in the case of Reddy et al. [29]. Linear neural network architectures such as these traditionally suffer from the vanishing gradient problem, whereby the size of the gradient is halved in rectified linear unit layer, as the network back-propagates up through the layers of parameters the size of the gradient decreases with each additional layer, effectively decreasing the effectiveness of backpropagation with each additional layer. This limits the useful depth possible with linear architectures in complex without significant augmentation [41, 42].

**Table 7:** Highest classification accuracy of study results in comparison to those previously published abdominal ultrasound studies

Author	Images	Sets	Cross sections	Model	Average accuracy (%)
Cheng and Malhi [26]	5518	185	11	CaffeNet (Alexnet)	77.30
				VGGNet (VGG-16)	77.90
Xu et al. [28]	187,219	706	11	ResNet50 (STL)	81.22
Reddy et al. [29]	1906	983	6	Alexnet	95.27
				VGG-16	97.37
				VGG-19	98.03
				GoogLeNet	96.49
				InceptionV3	97.89
				Resnet-18	97.37
				Resnet-50	98.77
				Resnet-101	98.24
This studies results	26,294	64	16	Alexnet	80.80
				VGG-16	82.59
				Resnet-50	83.48
				Resnet-101	82.14
				GoogLeNet	83.93
				InceptionV3	83.93

**Fig. 3:**
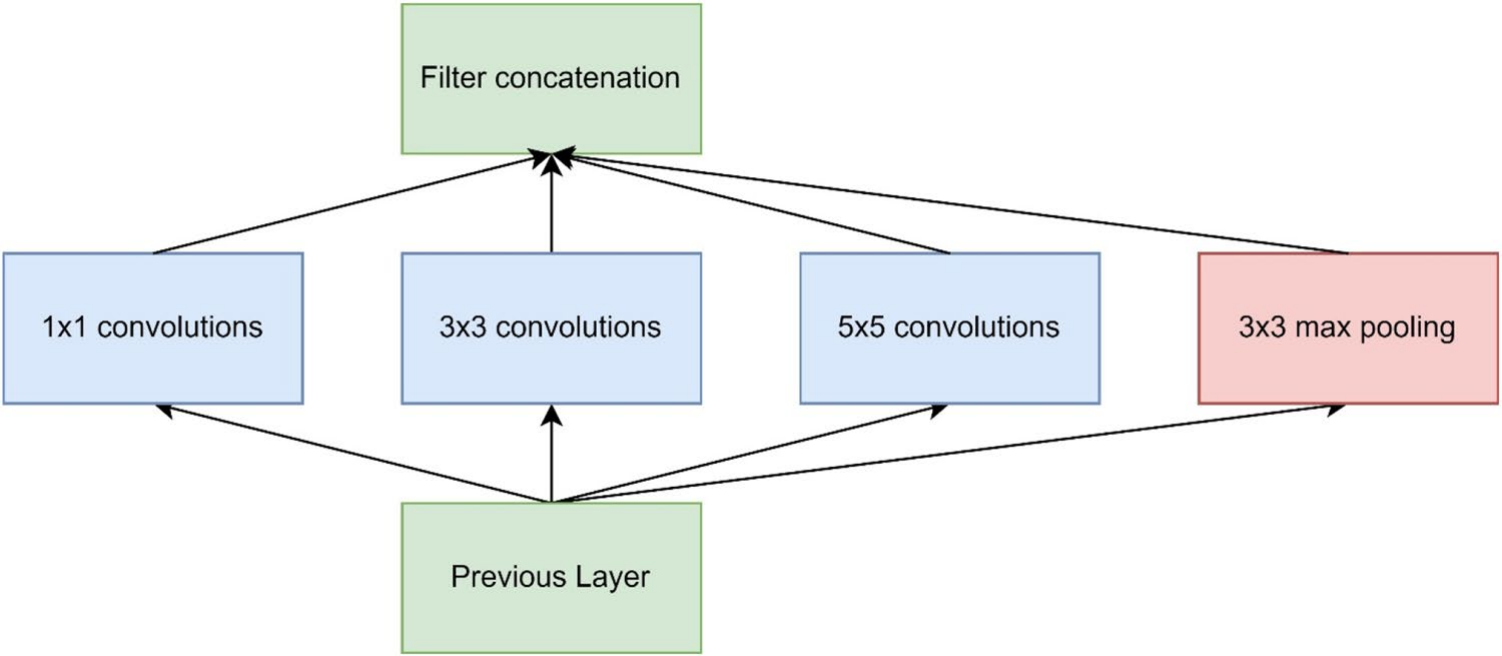
Example of an Inception module [38]

As in this study, cross sections containing overlapping landmarks and regions of interest such as the kidneys are shown to be a significant cause of classification error, in Cheng & Malhi [26] and Xu et al. [28] both transverse and longitudinal scans of the left and right kidneys cause significant additional classification error, Reddy et al. [29] while not containing multiple kidney classifiers, experienced similar error in liver cross sections where the right kidney appeared within the ultrasound image. A small reduction in accuracy can also be noted for larger scale Resnet networks in Reddy et al. [29] the resnet-50 achieved classification accuracy results 0.53% higher than that of the Resnet-101 compared to 1.34% in this study. While this would be expected in linear style networks, residual networks create feature maps of specific residual identifiers. These residual feature maps are propagated higher up the neural network with each training epoch effectively creating shortcuts within the model therefore reducing the effect of vanishing gradient [37]. Despite this, results suggest that standard ultrasound data may not have enough visual information to fully utilise networks larger than Resnet-50. The inception architecture uses a modular design approach to mitigate the vanishing gradient problem in GoogLeNet (Inception V1) [38] and InceptionV3 [39] convolution layers are clustered together into modules (as exampled in Fig. 3) instead of activated linearly. While more effective in this study, it did not achieve highest accuracy in Reddy et al. [29] where results were 2.28% lower for GoogLeNet and 0.88% lower for InceptionV3.

The results of this study are limited by the size of the test set of 14 patients, containing just 224 samples, necessary to ensure that no data leakage occurred during training. All patient sets are within normal range with no abnormal pathology or underlying conditions noted during ultrasound screening. All images were produced by a single machine, with all classification occurring at time of sampling by a single experienced operator. Only a single manually selected ideal plane image for each of the 16 plane categories was taken, while it would have been possible to take multiple samples from each patient set, there was insufficient differences to warrant including these results with a variance of less than 1% when the sample size was quadrupled.

## Conclusion

This study builds upon the current knowledge by evaluating the classification accuracy of three major neural network architectures using 16 upper abdominal ultrasound cross sections. Transfer learning using linear, residual and inception neural network architectures were all showed to be effective in classifying upper abdominal cross sections with the number of layers in the neural network being a less significant factor than the size of the dataset.

Applying neural networks to the recognition of cross-sectional abdominal imagery has much potential clinical significance, there networks could increase adherence to protocol by reducing scan variance due to user performance, both through assisting with sonographic training and through certifying that the region of interest has been fully captured for less experienced sonographers. It will allow experienced sonographers to put their full focus on the detection of anomalies while performing a required sweep with the neural network capturing the images mandated within the protocol automatically potentially reducing the time required to perform scans. Automatic capture of cross sections will also allow for better comparison in the case of surveillance scans in at risk populations and annual check-ups.

As neural network architectures further develop for image classification techniques it is important to continue to test their effectiveness on medical imaging such as ultrasound which provides more constrained visualisation data than that of traditional imagery. The study of neural networks for upper abdominal cross section classification has so far been limited, future works should examine the use of smaller networks potentially opening up use on mobile devices, as well as expanding the dataset size to allow for more effective training and validation. This should be achieved using methodologies that are cost effective [43].
